# Supplementary figures and images for: Fibroblast depletion reveals mammalian epithelial resilience across neonatal and adult stages
Source: J Cell Biol. Author manuscript; Available in PMC 2026 Jun 15. (PMC13267860; doi:10.1083/jcb.202507165)

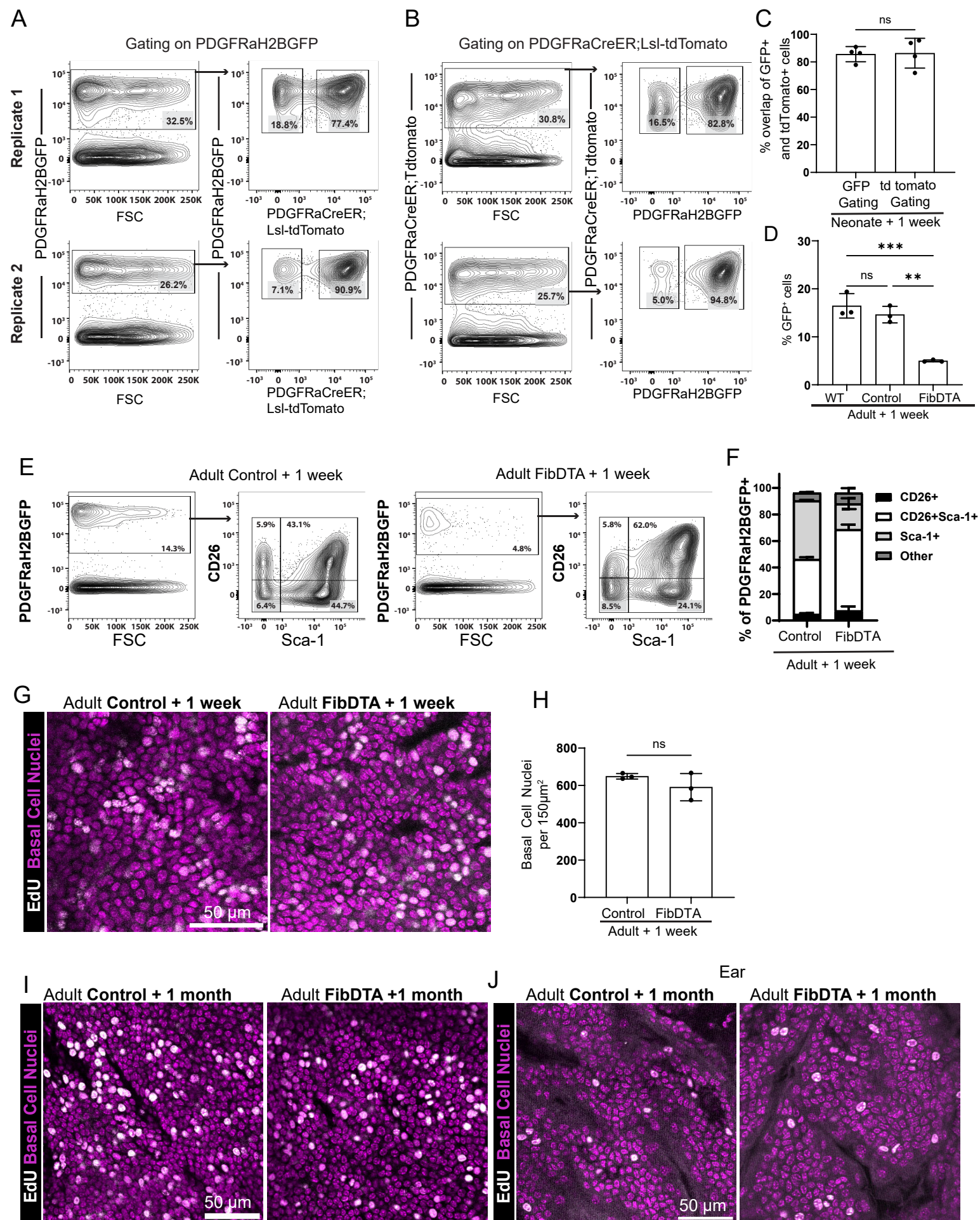

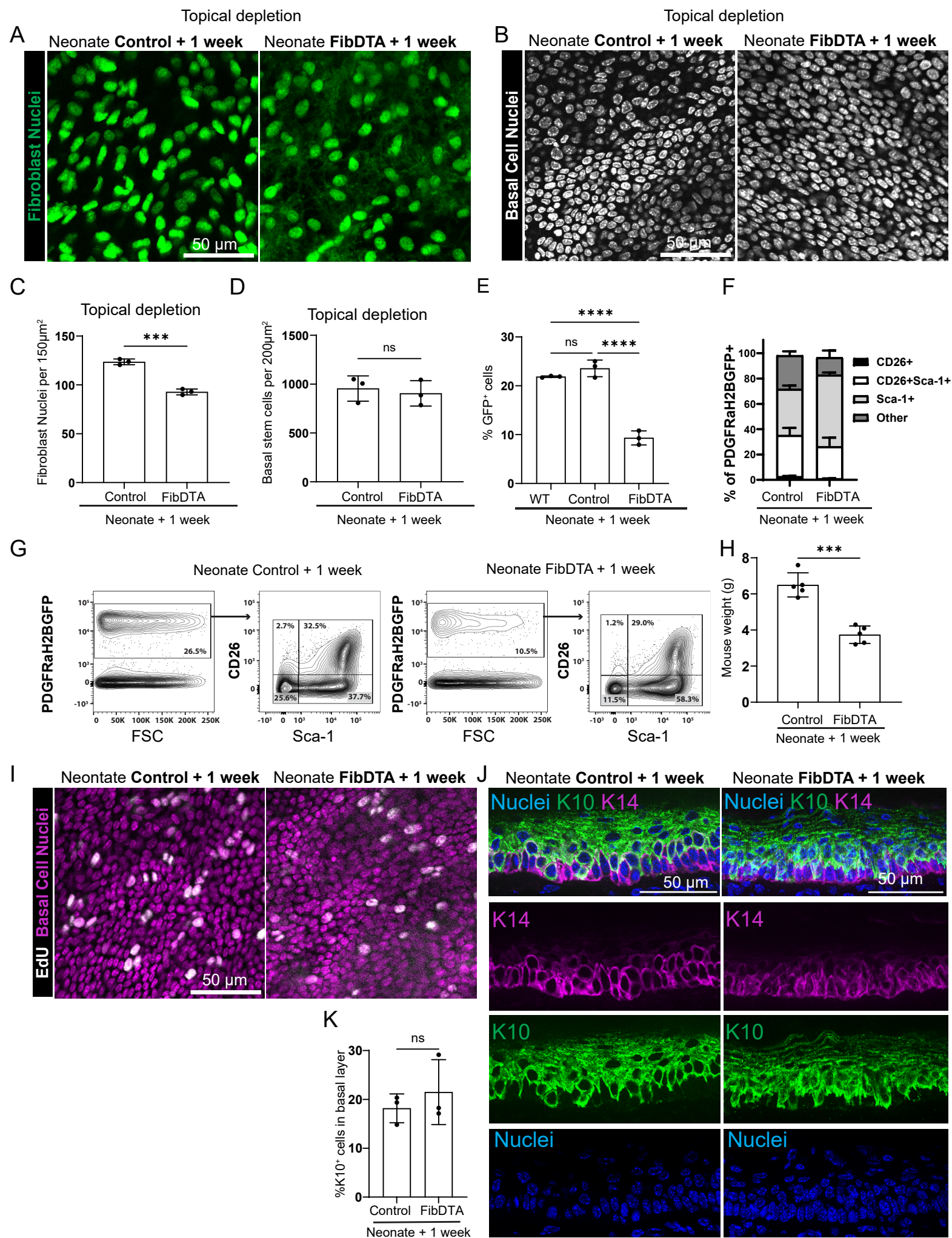

A

## Macrophages

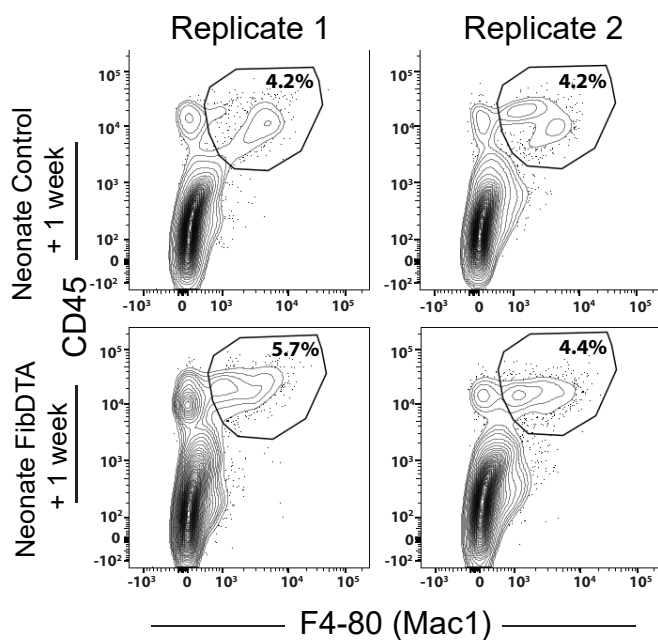

## B Neutrophils and Monocytes (of CD45+ cells)

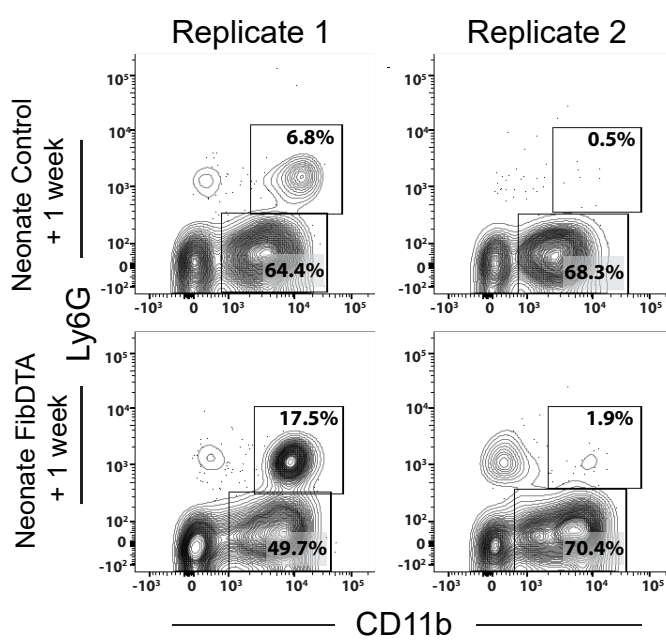

C

## T cells

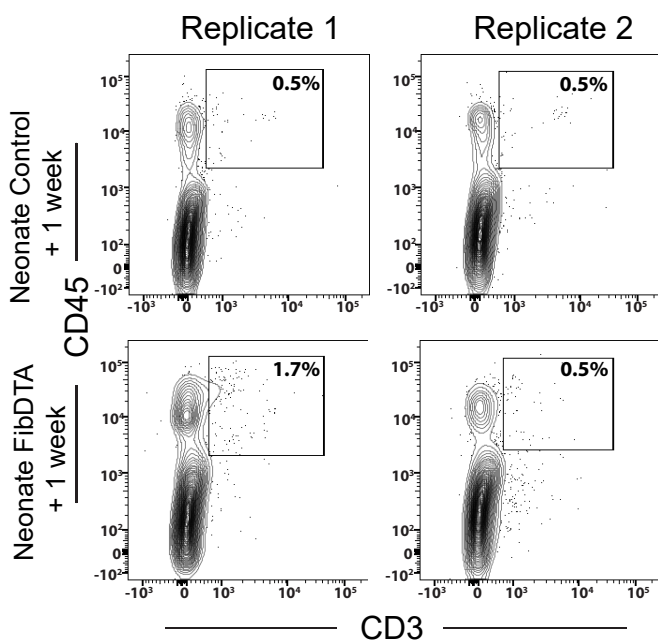

D

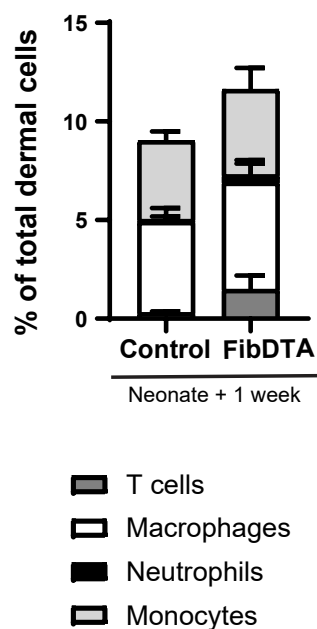

E

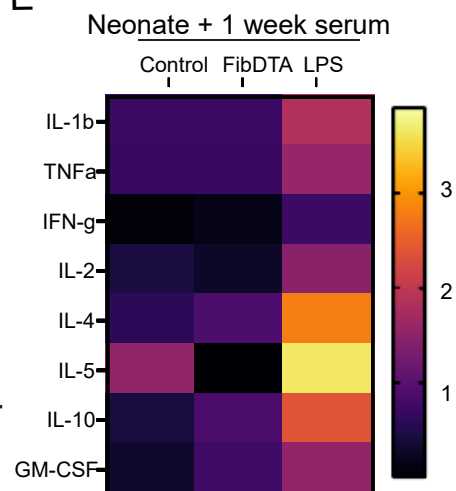

F

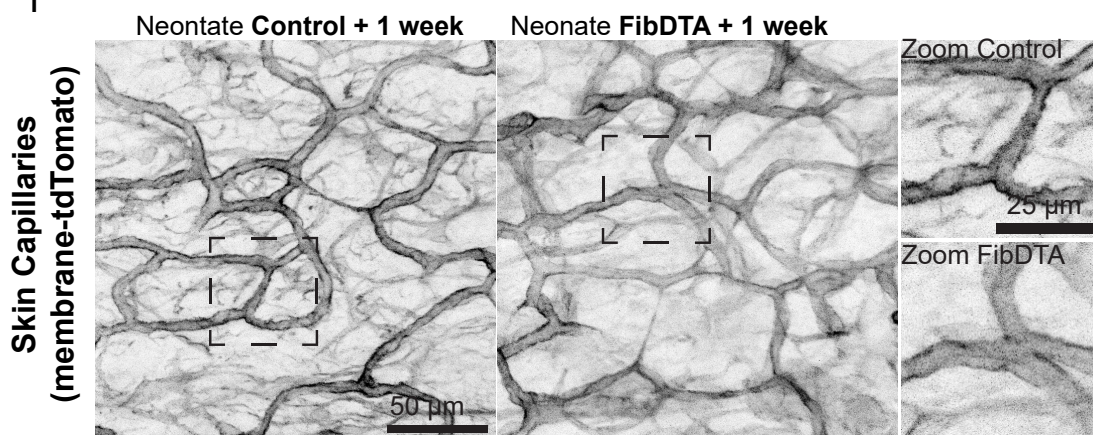

G

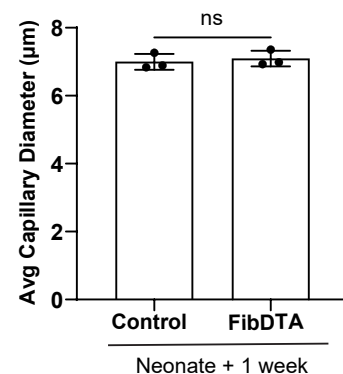

Supplement: Supplementary Figures S1-S3 — Supplementary Figure 1. (A) Representative FACS plots from two replicate neonatal mice one week post induction. Mice expressing PDGFRα-H2B-GFP; PDGFRα-CreER; lsl-tdTomato gating first on GFP+ cells and then tdTomato+ to determine the extent of the overlap (GFP+tdTomato+ dermal cells) between the two models. n = 4 mice. (B) Representative FACS plots from two replicates as in (A) expressing PDGFRα-H2B-GFP; PDGFRα-CreER; lsl-tdTomato gating first on tdTomato+ cells and then GFP+ to determine the extent of the overlap (GFP+tdTomato+ dermal cells) between the two models. n = 4 mice. (C) Quantification of the percent overlap of cells expressing both PDGFRα-H2B-GFP and PDGFRα-CreER; LSL-tdTomato, showing an average of 86% overlap regardless of the gating strategy. n= 4 mice p= 0.9098, ns, unpaired, two-sided Welch’s t-test. (D) Quantification of fibroblast depletion in adult mice one week post induction by FACS, comparing PDGFRα-H2B-GFP (WT), PDGFRα-H2B-GFP;lsl-DTA (Control) and PDGFRα-H2B-GFP; PDGFRα-CreER; lsl-DTA (FibDTA) mice. p = 0.4634, ns, *** p = 0.0005, **p = 0.0014. One way ANOVA with multiple comparisons. (E) Representative FACS plots showing fibroblast heterogeneity in adult mice one week post induction. n=3 control (PDGFRα-H2B-GFP; LSL-DTA) mice compared to n= 3 FibDTA (PDGFRα-H2B-GFP; PDGFRα-CreER ;LSL-DTA) mice after systemic depletion of PDGFRα+ cells. Cells were gated on GFP first, and then gated on CD26+Sca-1−, CD26+Sca-1+, CD26-Sca-1+ and CD26-Sca-11− (Other). (F) Quantification of fibroblast heterogeneity in adult mice one week post induction. n=3 control (PDGFRα-H2B-GFP; LSL-DTA) mice compared to n=3 FibDTA (PDGFRα-H2B-GFP; PDGFRα-CreER ;LSL-DTA) mice after systemic depletion of PDGFRα+ cells. p = 0.3183 ns (CD26+), p = 0.0524, ns (CD26+Sca-1+), **p=0.01 (CD26-Sca-1+), p=0.3954, ns (CD26-Sca-1−, Other), unpaired, two-sided, Welch’s t-test. (G) Representative whole mount images of control and FibDTA mice adult mice one week post induction after 6hr EdU [file NIHMS2179919-supplement-Supplementary_Figures_S1-S3.pdf]
